# Why is patient safety so hard in low-income countries? A qualitative study of healthcare workers’ views in two African hospitals

**DOI:** 10.1186/s12992-015-0096-x

**Published:** 2015-02-25

**Authors:** Emma-Louise Aveling, Yvette Kayonga, Ansha Nega, Mary Dixon-Woods

**Affiliations:** SAPPHIRE Group, Department of Health Sciences, University of Leicester, 22-28 Princess Road West, Leicester, LE1 6TP UK; Harvard T.H. Chan School of Public Health, 677 Huntington Ave, Boston, MA 02115 USA; Department of Welfare and Social Development, Catholic University of Rwanda, Huye, Rwanda; Institute of Public Health, University of Gondar, Gondar, Ethiopia

**Keywords:** Patient safety, Organization and delivery of healthcare, Developing countries, Africa, Resource-limited, Healthcare workers

## Abstract

**Background:**

The views of practitioners at the sharp end of health care provision are now recognised as a valuable source of intelligence that can inform efforts to improve patient safety in high-income countries. Yet despite growing policy emphasis on patient safety in low-income countries, little research examines the views of practitioners in these settings. We aimed to give voice to how healthcare workers in two East African hospitals identify and explain the major obstacles to ensuring the safety of patients in their care.

**Methods:**

We conducted in-depth, face to face interviews with healthcare workers in two East African hospitals. Our sample included a total of 57 hospital staff, including nurses, physicians, technicians, clinical services staff, administrative staff and hospital managers.

**Results:**

Hospital staff in low-income settings offered broadly encompassing and aspirational definitions of patient safety. They identified obstacles to patient safety across three major themes: material context, staffing issues and inter-professional working relationships. Participants distinguished between the proximal influences on patient safety that posed an immediate threat to patient care, and the distal influences that generated the contexts for such hazards. These included contexts of severe material deprivation, but also the impact of relational factors such as teamwork and professional hierarchies. Structures of authority, governance and control that were not optimally aligned with achieving patient safety were widely reported.

**Conclusions:**

As in high-income countries, the accounts of healthcare workers in low-income countries provide sophisticated and valuable insights into the challenges of patient safety. Though the impact of resource constraints and weak governance structures are particularly marked in low-income countries, the congruence between accounts of health workers in diverse settings suggest that the origins and solutions to patient safety problems are likely to be similar everywhere and are rooted in human factors, resources, culture and behaviour. While additional resources are essential to patient safety improvement in low-income settings, such resources on their own will not be sufficient to secure the changes needed.

**Electronic supplementary material:**

The online version of this article (doi:10.1186/s12992-015-0096-x) contains supplementary material, which is available to authorized users.

## Background

Though patient safety research and activity has traditionally been preoccupied with high-income countries (HICs), recent years have seen a shift of focus to low-income countries (LICs) [1-3]. A growing global policy emphasis is evident on the need not just to enable universal access to care, but to ensure that care “is of sufficient quality to be effective” and does not cause harm to patients [4,5]. Securing patient safety in practice remains a persistent challenge, however [6].

In high-income countries, the views of practitioners at the sharp end of health care provision (i.e. those directly involved in delivering care to patients) are increasingly valued in at least four ways: as a source of intelligence about defects that underlie risk in organisations and about the opportunities for improvement [7,8], as an indicator of quality of care [9,10], as a way of ensuring that patient safety interventions are most appropriately and effectively designed and implemented [11-13], and as a resource that enables the science of safety to develop [14]. A recent public inquiry into a scandal involving failings of patient safety at a hospital in England went so far as to recommend that staff feedback on quality of care be “not only encouraged but insisted upon” in healthcare organisations [15]. A subsequent report further recommended that “leaders need first-hand knowledge of the reality of the system at the front line, and they need to learn directly from and remain connected with those for whom they are responsible” [16].

While existing research in low-income countries suggests that healthcare workers express significant concern about the quality and safety of care [17,18], only limited research has examined the reasons for this. With some exceptions [17,18], this work has mostly focused on staff responses to particular patient safety initiatives conceived in terms of adherence and performance [19,20]. In contrast to high-income countries, the insights that these workers may offer regarding what affects patient safety have remained largely neglected. Moreover, the driving concepts and dominant tools in the patient safety field have, for the most part, been developed in the context of high-income country healthcare systems; it is not (yet) clear to what extent these conceptualisations align with the views and experiences of staff working in diverse socio-economic contexts.

In this article, we give voice to how healthcare workers in two East African hospitals identify and explain the major obstacles to ensuring the safety of patients in their care, and reflect on the insights their views offer for targeting improvement efforts in diverse contexts.

## Methods

We conducted semi-structured interviews with staff in two government referral hospitals in two countries in East Africa. At the request of study participants, the countries are not named. It should be noted, though, that both hospitals were operating in a context of growing local and national emphasis on quality and safety of healthcare, and, despite significant national resource constraints, important gains (such as progress towards Millennium Development Goals) have been made in recent years in these settings.

Purposive sampling of staff was undertaken in the departments that the two hospitals had chosen as the focus of their patient safety improvement efforts. Though our study did not seek directly to evaluate these efforts, these departments were an appropriate focus as staff would be more likely to be sensitised to issues of patient safety. Sampling aimed to include workers of differing grades, areas of practice, and management responsibility. Interviews were conducted, with informed consent from participants, in English or another locally used language, and covered perceptions of patient safety and challenges in delivering safe care (see Additional file 1 for a copy of the topic guide). Interviews were conducted by author ELA and author YK in one site, and by ELA and author AN in the other site. All interviews were digitally recorded (except for one where consent was not given to recording and notes were taken instead), transcribed and (where necessary) translated.

Data were analysed using the principles of thematic network analysis [21], supported by Nvivo software, and guided but not constrained by sensitising concepts derived from the research questions [22]. Data from both sites were combined, as the focus was on characterising common themes rather than differences. ELA analysed all data from both sites. Independently, YK and AN each analysed the data from one of the sites. All three researchers then discussed the themes identified from both sites; ELA integrated the analyses and all three agreed on the final set of themes, sub-themes and their interrelations.

Ethical approvals for the study were received from the University of Leicester Committee for Research Ethics, and the relevant national or institutional review boards in each of the African countries. We do not provide further detail of the hospitals (or the ethical approvals) that could make the participating hospitals identifiable. Details of interview participants are similarly limited.

## Findings

A total of 57 semi-structured, in-depth interviews were conducted (31 in one hospital, and 26 in the other). Participants included nurses and physicians from surgery and obstetrics-gynaecology departments, clinical services staff (e.g. pharmacy, microbiology), administrative staff and hospital management, of differing levels of seniority (Table 1). In one hospital we interviewed a small number of paediatric nurses; in the other, a small number of cleaning staff.

**Table 1:** **Number of interviewees by profession and by management responsibility**

**Profession**	***Number of interviewees***	**Management Responsibility**	***Number of interviewees***
Nurses (surgery, paediatric, gynaeco-obstetric departments)	21	Senior hospital management	3
Physicians (surgery, gynaeco-obstetric departments)	16	Head of Department	10
Anaesthetic technicians	8	Nurse in charge of department or unit	8
Clinical services (e.g. microbiology, pharmacy, blood bank)	10	No managerial responsibility (fully qualified)	29
Administration	2	Trainees	7
**TOTAL**	**57**	**TOTAL**	**57**

We start by describing how participants conceptualised ‘patient safety’. Participants’ accounts of the obstacles to securing safe care for patients are then presented in terms of the three major themes generated by the analysis: material context (including physical infrastructure, equipment and supplies); staff and staffing (including issues around the number and training of staff and staff retention); and relational context (inter-professional relationships including teamwork, hierarchical dynamics and the implications for governance and accountability).

Staff in these low-income settings offered an all-encompassing and broadly aspirational definition of patient safety, one that included the need to avoid harm to patients, and tended to emphasise quality as much as safety of care. These accounts are likely to have been influenced to some extent by ideas about patient safety from high-income countries that have begun to disseminate world-wide; the term “patient safety” was a relatively new one for some and was often used in English rather than translated into the local language. However, the underlying concept was understood by participants to be strongly aligned with their sense of a healthcare workers’ professional duty.

*So from diagnosis, surgery up to when the patient is discharged, and then follow-up, in all those steps something can go wrong, so for patient safety, you have to always be aware that anything can go wrong in between, so those things should be prevented. [033, Physician]*

Participants’ accounts were notable for how they did not attribute problems in delivering on this aspiration for high quality, safe care to any single cause; instead, multiple, inter-related challenges were described. Participants distinguished between the proximal influences on patient safety that posed an immediate, direct threat to patient care (such as non-availability of a surgeon to perform an emergency operation) and the distal influences that generated the contexts for such hazards (such as a national shortage of trained staff) (Table 2). Distal influences included contexts of severe material deprivation, but they also included the impact of relational factors such as teamwork and structures of accountability and management.

**Table 2:** **Examples of the distinction between proximal and distal obstacles to patient safety identified by staff**

**Proximal obstacles**	**Distal factors**
Shortage of skilled nursing staff (e.g. in operating theatres)	-Human resource shortages nationally
	-Few opportunities for specialist training or updating of skills
	-Human resource management policies
	-High turnover of staff, associated with staff dissatisfaction
Shortage of material resources (e.g. sterile drapes)	-Non-functional equipment due to lack of trained maintenance staff (e.g. broken autoclaves)
	-Infrastructure failures (lack of electricity to power autoclave or water supply to ensure reliable supply of sterilised equipment)
	-Lack of access to and/or budget for materials and parts (nationally and/or locally)
Lack of access to necessary drugs (e.g. antibiotics)	-Patient poverty
	-Lack of access to or budget for desired drugs at national level
	-Problems with storage and accessibility of drugs -Delays and other problems with procurement processes

### Material context: physical environment, equipment and supplies

All participants described how the material and physical context in which they worked profoundly affected their ability to provide safe care. They highlighted the poor condition of hospital buildings, including broken windows and malfunctioning doors, inappropriate building design, overcrowded clinical areas and difficulties in controlling human traffic. Poor infrastructure was widely reported: the electricity supply was unreliable, as was the water supply.

*I remember one case in the neonatal ward. The baby passed away because of shortage of electricity… he died because he didn’t get the oxygen he needed. The oxygen wasn’t working because it works with electricity. [017, nurse]*

*For example inside our theatre there are places you find structures which are old and are not replaced, which can be a source of infections. [037, nurse]*

*The maternity ward is disgraceful, you see? No beds, then patients lie on the floor, postpartum, and sometimes you don’t have beds to examine [patients on] [024, physician]*

Very high patient volumes, lack of dedicated space for emergencies and exacerbation of over-crowding by medical student observers further intensified the challenges.

*When all the [operating] rooms are occupied by elective patients and we receive an emergency case, we don’t have a free room to do emergency procedures. For this reason a mother whose baby is in foetal distress may lose its life. [015, nurse].*

Lack of materials and equipment were identified as major hazards. Some equipment was not available or there was not enough of it, and consumables (e.g. gauze, sterilised drapes) and drugs (e.g. antibiotics) were limited in range and in short supply.

*You cannot have patient safety or help the patient if you don’t have materials to use. [049, nurse]*

Staff attributed resource shortages to a number of factors. Some equipment, drugs or materials were simply not available for purchase or were not affordable within national budgets. Even where equipment was available, staff reported it was often not well maintained, and was unusable or unreliable. Staff blamed both the absence of skilled staff to maintain equipment and, to a lesser extent, careless or incorrect handling. The facilities for washing, drying and sterilising re-usable materials such as drapes were poor and affected by power cuts. Further supply problems arose because patients were required to make out-of-pocket contributions to the cost of care and the purchase of materials, yet the patient population suffered high levels of poverty.

*There were no sterilised packs … The problem was with the autoclave because the night shift workers were working all night and they used all of them. [052, nurse]*

*In our pharmacy we often have stock shortage: we may prescribe a medicine which the patient cannot find in our pharmacy, and then when he goes to a private pharmacy it is expensive, and this is a very poor patient population so he may delay waiting for money for three or four days. For a patient to wait two or three days without antibiotics, it affects his life and also it affects us. [037, nurse]*

Neither financial problems nor deficits in national availability were seen as the whole story by participants, however. In both sites, despite some recent improvements in hospitals’ budgets, staff reported weaknesses in national and local procurement systems and local distribution and storage of materials contributed to hazards.

*[The administration] is not running smoothly… You can find the money, but some materials are bought but kept in the stores, or they are not bought in a timely way. [033, physician]*

### Staff and staffing

A critically important feature of the context of material deprivation was low staffing levels and perceived deficits in the competences of staff. Participants’ accounts often emphasised the impact on patient safety of staff shortages, which they linked to national shortages of trained personnel. Times of particular risk included busy or emergency periods, night-shifts (when staffing levels were even lower), or when staff had to work additional or extra-long shifts (e.g. working the day shift following a night shift).

*The nurses are overloaded - they try their best, but we recognise that they are few, so such low numbers means that probably some patients on the ward will not be well managed. [048, physician]*

*The number of staff is not enough, to the extent that due to being few staff your job is always tiring. You do not get time to rest and you cannot give good treatment when you are tired. [049, nurse]*

Inadequate staffing levels were compounded by inadequate training and limited opportunities for specialised or on-going training, especially for specialist physicians, nurses and anaesthetic staff. For example, nurses did not receive any specialist training before beginning work in the operating theatres. Skills specific to patient safety, such as infection prevention and control, were inconsistently taught.

*It’s a problem, we have a shortage of staff here, and to implement the patient safety, as I saw it at [hospital in UK], they have a big team who are doing only that, but here in the whole hospital we only have one person [054, clinical service worker]*

High staff turnover (in particular wards and in the hospital as a whole) added to the problems, as did frequent rotation of nurses between departments. “Fresh” staff with little training in clinical specialities tended to be more prone to errors, and imposed a significant burden of education and induction on already over-stretched senior staff.

### Relational context: teamwork and hierarchy

The available staffing was reported to be compromised by poor teamwork or conflicts between different professionals. Although staff generally felt there was a good level of cooperation within professions (e.g. anaesthetists supporting each other), weak communication and coordination between professions, teams, different wards or departments was frequently described.

*Problems may be created, for example, sometimes they come with a patient who needs oxygen without telling us to prepare beforehand, it may take some time until we find the oxygen. [028, nurse]*

*Up to now it’s not at all organised, for example there isn’t good communication between lab staff and the hospital. [054, clinical service worker]*

Sometimes overt conflict was evident, often associated with emergency situations, shortages of space or materials, or staff who were inadequately trained for the job they were being asked to perform.

*The other [problem] is lack of tables, you know, we actually fight with the obstetric department [for the table]” [033, physician]*

Poor teamwork due to hierarchical dynamics was a major concern. Among staff who were not physicians, feelings of being disrespected or not being held in sufficient esteem were commonly reported. The salaries of these non-physician staff (set by national governments) were very low, especially compared with doctors, and they were offered few incentives (such as additional training opportunities or additional payment) to reward their hard work. Participants reported that the associated low morale and increased staff turnover had undesirable consequences for patient safety.

Hierarchical dynamics also contributed to elite groups – such as doctors – feeling that they could flout patient safety rules with impunity, since they did not recognise those “beneath them” (such as nurses and anaesthetists, who were not physicians) as having the authority to control or sanction their conduct. Their behaviours were reported to cause threats to patient safety, including, for example, increased infection risks and failure to act on concerns raised by colleagues.

*[Surgeons] don’t even listen to you [a nurse]. I usually tell them what should be done, but they don’t usually do it in practice, and they know what they mistakenly do […] They know what the standard should be, and you notice that they are the ones who make mistakes. [..] For example, in waste segregation, we have notices about where contaminated gowns and drapes should be placed, [but] they throw these things on the ground instead of putting them in their place, even though I remind them. [015, nurse]*

Structures of authority, governance and control that were not optimally aligned with achieving patient safety were widely reported. Managers were described as often limited in their ability to control what staff did, with the result that workers did not always follow protocols or complete their duty hours. According to the participants, transparency and predictability were largely absent, and lack of clarity was evident about what action would be taken by hospital management when staff did not fulfil their duties or to ensure that learning occurred following preventable errors.

*If somebody is not there in a duty hour, in the night time, in a ward, and a patient is affected, I don’t think it’s enough even to decrease his salary, maybe he should go to court […] but to do such a thing there is no law, you can’t do such a thing, so you start to become somewhat lenient, you know, so that creates an open point for others also to create a problem [009, physician]*

*I saw a [surgery] patient who came back because a pack had been forgotten inside…Nothing - I didn’t see any measures taken. [016, nurse]*

## Discussion

As studies from high-income countries would lead us to expect, these accounts of 57 healthcare workers across two hospitals in different low-income countries provide sophisticated insights into why patient safety is so hard, and affirm the importance of valuing the accounts of staff in all care settings. Participants offered aspirational definitions of patient safety that were similar to those used in high-income countries, and that were aligned with their sense of professional duty and their concern for the welfare and rights of their patients. They identified proximate challenges that had a bearing on patient safety at the sharp end. Material deprivations were strikingly prominent in their accounts; staff were working in degraded environments that constantly eroded the buffers of safety. But these accounts were also remarkable for how they demonstrated the relevance and deeply structuring effects of distal features of the national and institutional environments, including the budget allocation for government hospitals, national provision for training and remuneration of healthcare workers, and procurement processes.

By demonstrating a remarkable symmetry with the accounts of staff in high-income countries (which also feature concerns about material and human resource shortages, hierarchical dynamics and inadequate organisational systems [7]), these accounts suggest that the origins and solutions to patient safety problems are likely to be similar everywhere and are rooted in human factors, resources, culture and behaviour [23,24]. These findings are a corrective to the “uniqueness bias” [25], or assumption that there must be something exceptional and different about patient safety in low-income countries.

At the same time, what distinguishes these findings in African countries from high-income countries is not the nature of the hazards that threaten patient safety, but the scale and impact of the material deprivation and the relative weakness of structures of governance and accountability. Without denying the grim realities of poor resourcing, this work helps in clarifying where the targets for intervention should be, in so far as it suggests that – as in high-income countries – improving patient safety requires attention to not *only* to resource levels, but to culture and organisational systems [6].

There can be no doubt that continued investment in upgrading the material infrastructure of care will be essential to improvement; the deprived circumstances in which patients are being cared for directly and unambiguously undermine efforts to avoid harm. Without investment, little can be done about many of the problems that participants describe: hospitals that are too small and inadequately resourced yet experience overwhelming patient demand will always struggle to ensure patient safety. When staff feel there is little they can do to change their material, cultural and physical conditions, the result may be apathy and fatalism [26] with risk and poor outcomes being seen as unavoidable. Such fatalism may be further reinforced by the seeming insurmountability of the scale of problems faced [27] by staff who already feel over-burdened, under-paid and at times unfairly treated and insufficiently respected for their work. But our interviews also reveal the hard fallacy of assuming both that more money is all that is needed, and that nothing can be done to improve safety without extra financial investment.

Participants’ accounts made clear that, in addition to adequate materials and human resources at the sharp end, many of the basic skills and processes of hospital management require significant strengthening. Without improvements in more distal contributions to patient safety in areas such as procurement, distribution and equipment maintenance, use of the available resources is likely to remain sub-optimal – as high-income countries have also found [28]. Similarly, while the number of staff employed needs to be increased to cope with the high level of patient need, improved staffing levels on their own are not enough: staff in all settings need to be led, managed and well-supported [29]. The working conditions and morale of staff need as much attention as the physical and infrastructural conditions in which they work [17,24].

A further target for intervention made visible by our interviews, and one that may be achieved at relatively low financial cost, if not without significant effort, lies in the conduct of staff, their relationships with colleagues, and the decisions they make. As in high-income countries, justifications for poor care and ways of working with colleagues are influenced by the norms and values of professional contexts and efforts to claim (or retain) identities or status [14,30]. Hospitals in our study did not escape threats to patient safety associated with unhelpful hierarchies, poor teamwork and professional boundaries that have plagued hazardous industries everywhere [31]. Even where there were protocols nominally in place (e.g. for hygienic handling of clinical waste), anaesthetists and nurses sometimes struggled to secure compliance from the physicians who held higher status. When surgeons ignore nurses’ requests to put contaminated drapes in the allocated bins and instead throw them on the floor, they are not simply making technical judgements about infection risk; they are also making normative judgements about what is acceptable behaviour. These “cultural” aspects of patient safety may be hard to shift, but it can be done; again, they should be a focus of change efforts in all healthcare settings (not just low-income ones) [32].

This study does have limitations. It was conducted in two hospitals in two East African countries, and the extent to which our findings are generalizable cannot formally be tested within the scope of the current study. The chosen sites are, however, unlikely to be atypical, either in terms of their structural characteristics or their exposure to some patient safety and quality improvement thinking imported from high-income countries – for example, many hospitals are participating in programmes such as the WHO African Partnerships for Patient Safety programme and other internationally-supported initiatives, as well as Africa-based and national initiatives like the Council for Health Service Accreditation of Southern Africa (COHSASA) accreditation process.

## Conclusions

Few of the obstacles to patient safety identified by staff can be overcome without the right structures of governance, management and accountability in place, yet it was clear from our interviews that all of these need substantial improvement: optimising patient safety within the available resources will require engagement and leadership from government at all levels [33], not least to avoid reinforcing the sense amongst (demoralised) staff at the sharp end that they are being forced to shoulder responsibility for patient safety in a context which consistently undermines their efforts. The distal obstacles identified underscore the need for a multi-faceted approach to strengthening patient safety which focuses not only on the sharp-end, but also on organisational ‘fitness’ and institutional infrastructure at local and national levels. Additional resources are absolutely necessary to patient safety improvement in low-income settings, but listening to staff confirms that resources on their own will not be sufficient to secure the changes needed.

## Additional file

Additional file 1:
**Interview topic guide.**
