# Supplementary material for: Why is patient safety so hard in low-income countries? A qualitative study of healthcare workers’ views in two African hospitals
Source: Global Health. 2015 Feb 25;11:6. doi: 10.1186/s12992-015-0096-x (PMC4349795; doi:10.1186/s12992-015-0096-x)
Supplement: Additional file 1: — Interview topic guide. [file 12992_2015_96_MOESM1_ESM.docx]

**Additional File 1 - Basic Topic guide**

- What is your role, and how long have you been doing it?
- What do you LIKE about your job?
  - What do you dislike?
- What does your role involve?
- WHO do you work most closely with?
  - How would you describe your working relationship with nurses/ anaesthetists/ doctors/other
- What are the main challenges you encounter to delivering good care for patients?
- What sorts of mistakes / things go wrong most commonly?

***What does patient safety mean to you?***

- What do you think should be the main priorities for this hospital in terms of improving the quality and safety of services? What changes would you like to see?
- What does patient safety mean to you?
  - How does it relate to your work/ role as a XXXX
- What are the main obstacles to securing safe care for all patients?
- Has patient safety always been part of your role, or is it a new thing?
- To what extent do you believe patient safety is a priority for this hospital?
  - What makes you think so/ what evidence is there that PS is a valued priority?
- What kinds of things make it more or less difficult for you to prioritise patient safety in routine delivery of care?
- If a relative of yours was being admitted to this hospital, what would you be most concerned about?
- What would you describe as the strengths of the hospital/ the care provided to patients?

***Patient safety interventions***

- Have you been involved in any patient safety interventions in this hospital, and in what way?
- How was this intervention introduced?
- Can you tell me about this initiative – what are its goals and what does it involve?
- What kind of impact do you think this initiative has had? (positive or negative)
- What have been challenges in making change?
- Is there anything you would like to add?
- Do you have any other questions?

Thank you for your time
